# Psychometric properties of a DTD self-report scale among Portuguese adolescents from the community

**DOI:** 10.1080/20008066.2026.2742128

**Published:** 2026-10-30

**Authors:** Diogo Morgado, Ricardo Pinto, Julian D. Ford, Angela Maia

**Affiliations:** a School of Psychology, University of Minho, Braga, Portugal; b Faculty of Psychology, University of Lusófona, Oporto, Portugal; c HEI-Lab: Digital Human-Environment Interaction Lab, Faculty of Psychology, University of Lusófona, Porto, Portugal; d Department of Psychiatry, University of Connecticut School of Medicine, Farmington, CT, USA

**Keywords:** Developmental trauma disorder, validation, self-report, adolescents, psychometrics, Trastorno por trauma del desarrollo, validación, autoinforme, adolescentes, psicometría

## Abstract

**Background:** Developmental Trauma Disorder (DTD) has been proposed as a developmentally informed framework to describe the pervasive dysregulation associated with chronic interpersonal trauma in childhood. DTD has been assessed by a validated structured interview, but no study has validated a self-report measure of DTD symptoms or examined its factorial structure in community adolescent samples. In addition, measurement invariance across sex has not been investigated, despite evidence of sex differences in trauma exposure and trauma-related symptoms.

**Objective:** To evaluate the psychometric properties of a self-report measure of DTD symptoms in a community sample of Portuguese adolescents by testing factorial structure, internal consistency, convergent and discriminant validity, and measurement invariance across sex.

**Methods:** A school-based sample of 444 adolescents aged 11–16 years completed a self-report DTD symptom scale and other theoretically related measures. Confirmatory factor analyses compared alternative models of DTD symptom organisation. Measurement invariance across sex was tested using the best-fitting model. Convergent and discriminant validity were examined using Pearson correlations and group comparisons based on trauma exposure criteria.

**Results:** A second-order model with three first-order factors provided the best fit. Configural and metric invariance across sex were supported, with partial scalar invariance after freeing two intercepts. Internal consistency was good to excellent, and evidence for convergent and discriminant validity was observed.

**Conclusions:** The findings provide the first evidence that DTD symptoms can be assessed using a self-report measure with acceptable factorial validity, reliability, and cross-sex comparability in a community adolescent sample.

## HIGHLIGHTS

- This study provides the first validation of a self-report measure of Developmental Trauma Disorder symptoms in a community adolescent sample.
- A second-order model with three first-order factors was supported, with evidence of measurement invariance across male and female adolescents.
- The measure showed good to excellent internal consistency and evidence of convergent and discriminant validity.

## Abstract (es)

**Antecedentes:** El Trastorno por Trauma del Desarrollo (DTD, por sus siglas en inglés) se ha propuesto como un marco conceptual que incorpora una perspectiva del desarrollo para describir la desregulación generalizada asociada al trauma interpersonal crónico en la infancia. El DTD se ha evaluado mediante una entrevista estructurada validada; sin embargo, ningún estudio ha validado una medida de autoinforme de los síntomas del DTD ni ha examinado su estructura factorial en muestras comunitarias de adolescentes. Además, no se ha investigado la invarianza de medición entre sexos, a pesar de la evidencia existente sobre diferencias entre sexos en la exposición al trauma y en los síntomas relacionados con éste.

**Objetivo:** Evaluar las propiedades psicométricas de una medida de autoinforme de síntomas de DTD en una muestra comunitaria de adolescentes portugueses, mediante el análisis de la estructura factorial, la consistencia interna, la validez convergente y discriminante, y la invarianza de medición según el sexo.

**Métodos:** Una muestra escolar de 444 adolescentes de entre 11 y 16 años completó una escala de autoinforme de síntomas de DTD y otras medidas teóricamente relacionadas. Los análisis factoriales confirmatorios compararon modelos alternativos de organización de los síntomas del DTD. Se evaluó la invarianza de la medición entre sexos utilizando el modelo de mejor ajuste. La validez convergente y discriminante se examinó mediante correlaciones de Pearson y comparaciones entre grupos basadas en criterios de exposición al trauma.

**Resultados:** Un modelo de segundo orden con tres factores de primer orden proporcionó el mejor ajuste. Los resultados respaldaron la invarianza configural y métrica según el sexo, y la invarianza escalar parcial tras liberar dos interceptos. La consistencia interna osciló entre buena y excelente, y se observaron evidencias de validez convergente y discriminante.

**Conclusiones:** Los hallazgos aportan la primera evidencia de que los síntomas del DTD pueden evaluarse mediante una medida de autoinforme que presenta una validez factorial, una fiabilidad y una comparabilidad entre sexos aceptables en una muestra comunitaria de adolescentes.

Exposure to traumatic experiences that are chronic, repeated, and interpersonal in nature is a prevalent and consequential public health concern in childhood and across the lifespan (Li et al., 2025; Madigan et al., 2025; Patel et al., 2025; Yearwood et al., 2021). Such exposure is associated with a wide range of emotional, behavioural, relational, and neurobiological sequelae that often persist across development and are linked to elevated risk for psychiatric comorbidity, functional impairment, and adverse life outcomes (D’Andrea et al., 2012; Morelli & Villodas, 2022). Although posttraumatic stress disorder (PTSD) provides a well-established framework for understanding trauma-related psychopathology, increasing evidence indicates that PTSD criteria do not adequately capture the breadth and developmental complexity of difficulties observed in youth exposed to chronic interpersonal trauma, particularly in domains of self-regulation, identity, and interpersonal functioning (Ford et al., 2018; van der Kolk, 2005).

## 1. Developmental Trauma Disorder and a transdiagnostic framework

In response to these limitations, Developmental Trauma Disorder (DTD) was proposed as a developmentally informed diagnostic construct designed to complement and extend PTSD in childhood and adolescence (D’Andrea et al., 2012; van der Kolk, 2005). DTD conceptualises trauma-related psychopathology as a pattern of pervasive dysregulation across multiple domains of functioning, rather than as a circumscribed set of fear-based symptoms. Specifically, DTD is organised around three core domains: (a) affective and physiological dysregulation, (b) cognitive and behavioural dysregulation, and (c) self and relational dysregulation, in the context of exposure to interpersonal victimisation and disruptions in attachment relationships with primary caregivers (Ford et al., 2018).

DTD is explicitly framed as a transdiagnostic construct, integrating symptom domains that cut across traditional internalising and externalising disorders. This framework aims to account for the high levels of psychiatric comorbidity observed following complex trauma by identifying shared developmental mechanisms – such as emotion regulation deficits, impaired executive control, and disturbances in self-concept and relationships – rather than attributing difficulties to multiple independent diagnoses (D’Andrea et al., 2012; Morelli & Villodas, 2022). In this respect, DTD overlaps conceptually with the ‘disturbances in self-organization’ (DSO) component of ICD-11 Complex PTSD (CPTSD), while remaining specifically grounded in childhood-onset trauma and developmentally anchored patterns of dysregulation (Haselgruber et al., 2020).

## 2. Empirical status and measurement challenges

Empirical investigations of DTD have provided preliminary support for its reliability, construct validity, and clinical utility, particularly in demonstrating that DTD symptoms as assessed by the DTD semi-structured interview are associated with psychosocial impairment above and beyond PTSD and cumulative trauma exposure (Ford et al., 2018, 2022). A recent systematic review concluded that studies largely support DTD as a coherent and potentially clinically informative construct, while also emphasising that the evidence base remains limited by methodological heterogeneity, small samples, and a lack of standardised assessment instruments (Morelli & Villodas, 2022).

A major limitation identified in the literature concerns measurement. To date, most empirical work has relied on a clinician-administered interview (i.e. the DTD Semi-Structured Interview), proxy indicators derived from existing symptom scales, or clinician ratings not specifically designed to operationalise DTD criteria (Ford et al., 2018, 2022; Morelli & Villodas, 2022). There is currently no widely validated self-report instrument for assessing DTD symptomatology in adolescents, despite the centrality of self-report methods in large-scale epidemiological research, school-based screening, and community mental health assessment. This gap restricts the capacity to test DTD in non-clinical populations, to conduct cross-cultural research, and to evaluate its dimensional properties using modern psychometric methods.

## 3. The need for community-based and cross-cultural validation

Most existing DTD studies have been conducted in clinical or high-risk samples, primarily in North American contexts. However, if DTD is to be conceptualised as a developmentally grounded, transdiagnostic framework for trauma-related psychopathology, its measurement properties must be examined in community samples and across cultural contexts. Adolescence represents a particularly relevant developmental period, as regulatory capacities, identity processes, and relational patterns undergo substantial reorganisation, and trauma-related dysregulation may become more differentiated and measurable through self-report (D’Andrea et al., 2012; Ford et al., 2018).

Cross-cultural validation is also essential. Cultural norms shape emotional expression, self-concept, and interpersonal functioning (Bovey et al., 2025), all of which are central to the DTD construct. Without evidence of validity in non-English-speaking populations, the generalizability of DTD remains uncertain.

## 4. Adolescence as a critical developmental epoch

Adolescence is a transitional developmental epoch involving major changes in brain functions related to cognitive, affective, interpersonal, and behavioural self-regulation, and along with increased sensitivity to peer influences that can interfere with those essential self/interpersonal regulation capacities (Ahmed et al., 2015; Blum et al., 2015; Stephanou et al., 2016). As a result of these changes, adolescence is a time of vulnerability to the adverse effects of prior or current exposure to interpersonal trauma that are the self-regulatory impairments described by the symptoms of DTD (Ford, 2017). Additionally, internalising problems that begin in early childhood may initiate a trajectory of chronic psychopathology that includes externalising problems in adolescence (Wiggins et al., 2015), thus resulting in the combination of internalising and externalising symptoms that are the hallmark of DTD.

## 5. The present study

The present study addresses these gaps by examining the psychometric properties of a self-report measure of Developmental Trauma Disorder (DTD) symptoms in a community sample of Portuguese adolescents. Consistent with the theoretical conceptualisation of DTD as a developmentally grounded and multidimensional construct (Ford et al., 2018; van der Kolk, 2005), the study evaluates whether DTD symptom domains can be reliably assessed via self-report and whether they demonstrate evidence of construct validity in a non-clinical population. Establishing valid self-report measures is essential for large-scale epidemiological research, school-based screening, and cross-cultural investigations, where clinician-administered interviews are often not feasible.

Specifically, the present study examines the internal structure of the scale by testing alternative confirmatory factor analytic (CFA) models reflecting different conceptualizations of DTD symptom organisation, including a unidimensional model, a correlated three-factor model corresponding to the proposed domains of affective/physiological, cognitive/behavioural, and self/relational dysregulation, a second-order model in which these domains load onto a higher-order DTD factor, and a hybrid model informed by DSM-5 PTSD symptom structure. Because previous studies have primarily relied on interview-based assessment and have not evaluated self-report measures in community samples, the relative fit of these competing models was examined in a partly exploratory manner. However, consistent with the theoretical formulation of DTD as a multidomain construct, multidimensional models were expected to provide a better fit than a unidimensional solution.

In addition, the present study evaluates measurement invariance across sex. Demonstrating invariance is necessary to ensure that the construct has the same meaning in male and female adolescents and that comparisons across groups are psychometrically valid. Because no prior study has examined measurement invariance of DTD symptoms across sex, this analysis was conducted in an exploratory but theory-informed manner, with the expectation that at least configural and metric invariance would be supported.

Construct validity was examined through tests of convergent and discriminant validity. Based on prior research indicating that DTD symptoms are associated with trauma exposure, posttraumatic stress symptoms, and emotion dysregulation (D’Andrea et al., 2012; Ford et al., 2018, 2022; Morelli & Villodas, 2022), moderate to strong positive associations with these constructs were expected. In addition, adolescents exposed to both interpersonal victimisation and attachment-related trauma were expected to report higher levels of DTD symptoms than those exposed to only one type of trauma. Discriminant validity was examined by testing associations with resilience, which was expected to show weaker and negative correlations with DTD symptoms, indicating that the scale captures trauma-related dysregulation rather than general psychological functioning. Finally, the total DTD scale and its symptom clusters were expected to demonstrate adequate to excellent internal reliability.

By validating a self-report measure of DTD in a non-clinical adolescent population, this study aims to contribute to the empirical evaluation of DTD as a developmentally informed, transdiagnostic framework for understanding the sequelae of complex trauma. Establishing reliable and valid assessment tools is a necessary step toward determining the construct’s applicability in epidemiological research, cross-cultural contexts, and prevention-oriented mental health practice.

## 6. Methods

### 6.1. Participants

The total sample was 444 adolescents. The sample included 191 (43%) males and 253 (57%) females. The mean age of the sample was 13 years (*M* = 13.14, *SD* = 1.04), ranged between 11 and 16 years old. All participants were 7th, 8th, and 9th grade students in public basic or secondary schools. Regarding nationality, 381 (85.8%) were Portuguese, and 66 (14.2%) reported another nationality (e.g. 50 were Brazilian, four were Angolan; two were Luso-French, one was Argentinian, Venezuelan, Chinese, Spanish, Italian; Luso-Angolan; Luso-Colombian). Regarding ethnoracial identification, most students self-identified as White (*n* = 396, 89.2%). Smaller proportions identified as Black (*n* = 22, 5.0%), Roma/Gypsy (*n* = 3, 0.7%), or Asian (*n* = 2, 0.5%). Additionally, 21 participants (4.7%) selected the ‘Other ethnicity’ option and provided open-ended responses using culturally specific Portuguese terms (e.g. *parda*, *morena*, *mulata*), which were translated literally for reporting purposes. Two thirds (*n* = 303, 68.2%) were from two-parent families, 81 (18.2%) were from single-parent families, 59 participants (13.3%) reported alternating residence between households, and one (0.2%) was in an institution.

Family monthly income ranged from 750 euros or less (*n* = 28, 6.3%) to 751–2000 euros (*n* = 152, 34.3%) and more than 2000 euros (*n* = 54, 12.2%), with 210 participants (47.3%) reporting that they did not know their family income.

Regarding paternal education, 140 participants (31.5%) reported that the father did not complete secondary education, 103 (23.2%) completed secondary education, 87 (19.6%) had a university degree, and 114 (25.7%) did not know the father’s level of education. Regarding maternal education, 103 participants (23.2%) reported that the mother did not complete secondary education, 117 (26.4%) completed secondary education, 135 (30.4%) had a university degree, and 89 (20.0%) did not know the mother’s level of education.

## 7. Measures

**The Traumatic Events Screening Inventory – Child/Self** (TESI; Daviss et al., 2000; Portuguese version Correia-Santos et al., 2018) is a self-report measure that assesses exposure to potentially traumatic events. This measure includes ‘noninterpersonal’ traumatic events, such as, accidents, illness, or natural disaster, as well as ‘interpersonal’ traumatic events, such as, physical abuse or assault, family violence, community violence, sexual abuse, caregiver impairment, neglect or emotional abuse. Sample items include:

Have you ever been in a really bad accident where someone could have been (or actually was) badly injured or killed? (like a bad car or bike crash, a fall, a fire, bad burn, almost drowning, or a bad sports injury),

‘Have you ever been separated from someone who you depend on for love or security for more than a few days?’, ‘Have you ever seen or heard people in your family physically fighting, hitting, slapping, kicking, or throwing things at each other?’. This scale has demonstrated test–retest reliability and interrater reliability (Daviss et al., 2000), as well as convergent and discriminant validity (Ford et al., 2000).

**Stress Coping Strategies Questionnaire** (SCSQ; Ford et al., 2015) is a 25 item self-report measure that assesses symptoms of DTD. Specifically, the instrument evaluates 3 clusters of DTD: emotion/somatic (8 items), attentional/behavioural (7 items), and interpersonal/self-dysregulation (10 items) (Figure 1 displays the SCSQ with the items labelled to show the 15 symptoms of DTD, e.g. B1, C1, D1). Participants rate the frequency that they use each coping strategy using a 4-point Likert scale, with 0 meaning ‘none’, and 3 meaning ‘most days’, yielding a total score ranging from 0 to 75, which indicates DTD symptom severity. Examples of items are ‘If you felt scared, mad, sad, or frustrated, how often did these feelings get so big that you blew up or just totally shut down’ (cluster B), ‘How often did you hurt your body on purpose because you felt bad or because it helped you vent or feel better for a while?’ (cluster C); or ‘How often did you attack someone because they were being unfair or disrespectful or mean or trying to hurt you or someone else?’ (cluster D).

**Figure 1.**
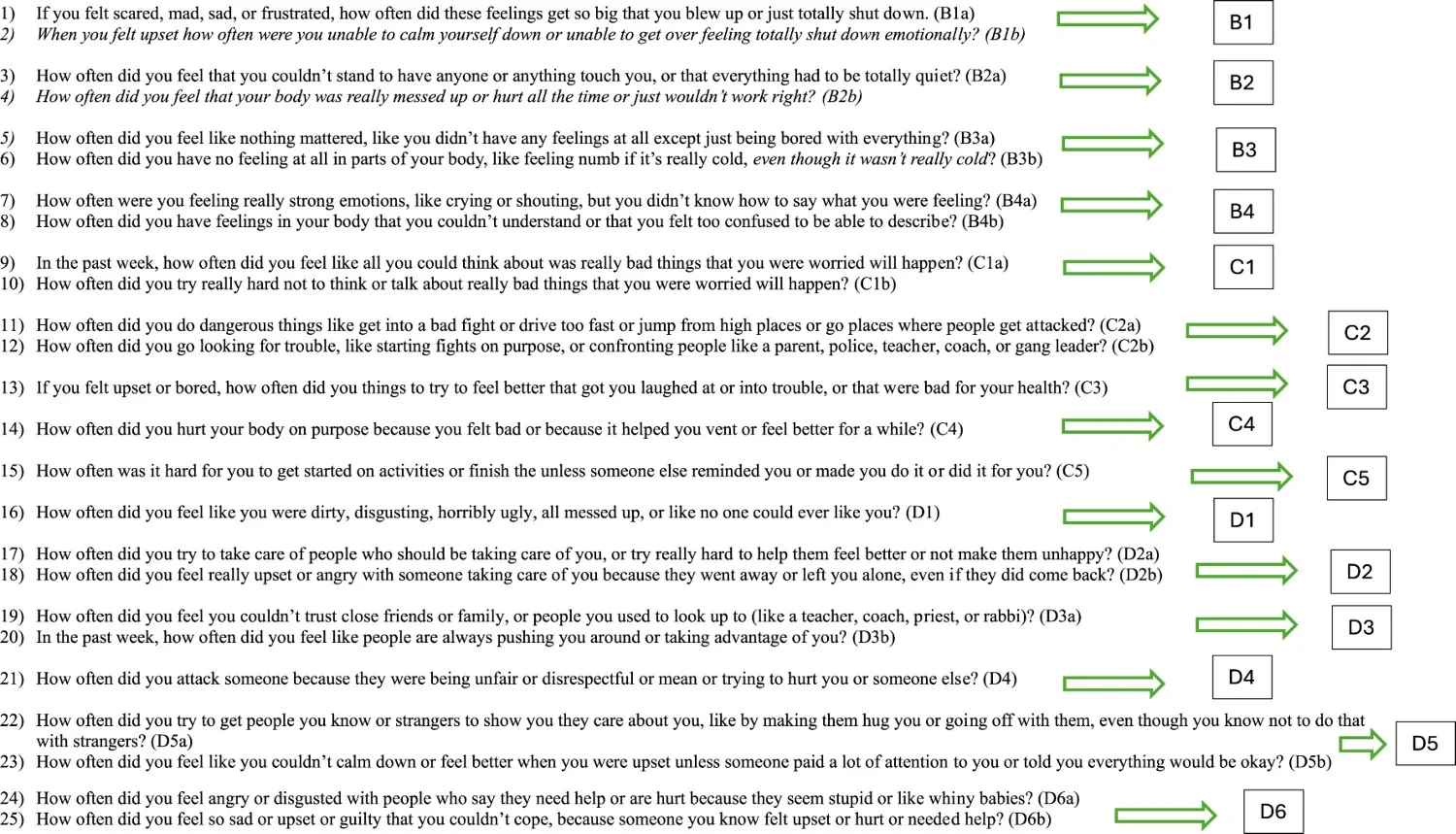
SCSQ with the items labelled to show the 15 symptoms of DTD, e.g. B1, C1, D1.

This questionnaire also includes two additional items. Item 26 asks whether the participant has experienced these problems at least some of the time during the past month, to which the response is dichotomous (yes/no). Item 27 assesses functional impairment, which is rated dichotomously across six domains: (1) make or keep friends; (2) get along with other kids your age; (3) do schoolwork; (4) get along with your teachers; (5) get along with people you live with; and (6) get your chores or other work done.

In accordance with guidance provided by the original authors (Ford et al., 2015), several items of the SCSQ include paired subitems that represent alternative behavioural manifestations of the same underlying DTD symptom. These paired subitems (e.g. B1a and B1b; B2a and B2b) are therefore scored as a single item by selecting the highest frequency endorsement across the two subcomponents, consistent with the instrument’s conceptual design. This scoring rule reflects the intention of the measure to capture symptom presence regardless of its specific behavioural expression. Items that are not subdivided (e.g. C4, C5, D1, D3) remain unchanged and are scored individually. Following this procedure, the 25 questionnaire entries correspond to 15 final scored items.

DTD criterion A was assessed through the TESI. At least one ‘interpersonal’ potentially traumatic event involving direct or witnessed victimisation (Criterion A1, e.g. abuse, family or community violence) and one ‘interpersonal’ potentially traumatic event involving disruption of primary attachment security (Criterion A2, e.g. traumatic neglect, death of or separation from a primary caregiver) must be endorsed for criterion A to be applied.

The original DTD questionnaire was translated to Portuguese by two independent researchers who were experts on the topic of traumatic stress. These two translated versions were compared and discussed to achieve an agreed standardised version. Facial validity was then assessed: the consensual version was tested in a pilot sample of eight adolescents to assess their comprehensibility of the items. This sample comprehended all translated items of the questionnaire. Finally, the Portuguese version was re-translated into English by two bilingual experts and was sent to the original author. Without proposals for change, this final version was approved by the original author.

**The International Trauma Questionnaire – Child and Adolescent version** (ITQ-CA; Cloitre et al., 2018) is a self-report measure that assesses symptoms of CPTSD. The ITQ-CA consists of six questions that evaluate symptoms of ICD-11 PTSD, and six questions that evaluate symptoms of DSO. In this measure, ICD-11 PTSD presents three clusters, where each cluster presents two symptoms. This is also the same for the DSO. Sample items for PTSD include ‘bad dreams reminding me of what happened’ (re-experiencing cluster), ‘trying not to think about what happened’ (avoidance cluster), or ‘being jumpy’ (sense of threat cluster). For DSO, sample items include ‘not being able to have feelings or feeling empty inside’ (affective dysregulation cluster), ‘thinking I am not a good person’ (negative self-concept) or ‘not feeling close to other people’ (disturbed relationships cluster). All items are scored on a 5-point Likert-scale ranging from 0 (‘not at all’) to 4 (‘almost always’). A score of 2 (‘sometimes’) or more on any item is taken to reflect the presence of a symptom. Functional impairment is rated dichotomously across a total of 5 domains: (1) getting along with friends, (2) getting along with family, (3) school-functioning, (4) other important areas of life (hobbies and other relationships), and (5) distress (worded as ‘general happiness’). A probable diagnosis of PTSD is assigned upon fulfilment of 1 symptom in each cluster and fulfilment of functional impairment, and a probable diagnosis of CPTSD is assigned upon fulfilment of 1 symptom in each cluster of DSO and functional impairment in addition to PTSD. This scale has shown internal reliability (Cloitre et al., 2018). The ITQ-CA scale showed good internal consistency in previous studies with youth samples (e.g. Kazlauskas et al., 2020). The internal consistency of the total symptom score for the scale in the present study was .91.

**The Child PTSD Symptom Scale – V** (CPSS-V; Gillihan et al., 2013; Portuguese version of Pinto et al., 2019) is a self-report measure that assesses the severity of PTSD symptoms in children and adolescents after having been exposed to a potential traumatic event. The instrument includes 20 items corresponding to PTSD symptoms according to the criteria of the DSM 5. Participants rate the frequency that they experience each symptom using a 4-point Likert scale, with 0 meaning ‘never’, and 4 meaning ‘6 or more times per week/almost always’, yielding a total score ranging from 0 to 51, which indicates PTSD symptom severity. Examples of items are ‘Having feelings in your body when you think about or hear about the event’ or ‘Trying not to think about, talk about, or have feelings about the event’. The child or adolescent is asked to think about the worst event that they experienced in their life when answering the questions. The study of the original scale demonstrated good to excellent internal consistency (Gillihan et al., 2013). The internal consistency for the present sample was .95.

**The Difficulties in Emotion Regulation Scale – Short Form** (DERS-SF; Kaufman et al., 2016; Portuguese version Moreira et al., 2022) is a self-report measure that assesses difficulties in emotion regulation. This scale presents 18 items that load onto six subscales: (1) Nonacceptance of Emotional Responses (e.g. ‘When I’m upset, I feel guilty for feeling that way’), (2) Difficulties Engaging in Goal-Directed Behavior (e.g. ‘When I’m upset, I have difficulty concentrating’), (3) Impulse Control Difficulties (e.g. ‘When I’m upset, I become out of control’), (4) Lack of Emotional Awareness (e.g. ‘I pay attention to how I feel’), (5) Limited Access to Emotion Regulation Strategies (e.g. ‘When I’m upset, it takes me a long time to feel better’), and (6) Lack of Emotional Clarity (e.g. ‘I am confused about how I feel’). Each subscale has three items, which are answered on a five-point Likert scale that ranges from 1 (almost never) to 5 (almost always). The subscale scores consist of the mean of the items, with higher scores indicating more difficulties in regulating emotions. Items from the Awareness subscale are reverse coded. The original scale showed excellent internal consistency, good test-retest consistency and good construct and predictive validity (Kaufman et al., 2016). The internal consistency of the total scale in the present study was .91.

**Child and Youth Resilience Measure** (CYRM-28; Lienbenberg et al., 2012; Portuguese version by Costa, 2019) is a self-report measure with 28 items that assesses resilience in children and adolescents. The resilience is measured through a 5-point Likert scale, ranging between 1 ‘totally disagree’ and 5 ‘totally agree’. The CYRM-28 scale is divided into three subscales, namely: Individual Factors, Caregiver Relationships, and Contextual Factors. The Individual Factors scale comprises three subscales: Personal Skills; Peer Support, and Social Skills. The Caregiver Relationships scale includes two subscales: Physical Care and Psychological Care. The Contextual Factors scale consists of three subscales: Spiritual, Educational and Cultural. In the original study, the total scale presented an alpha of .88. In this study, the internal consistency was .96.

### 7.1. Procedures

Ethical approval for this study was obtained from the Ethics Committee for Research in Social and Human Sciences of the University of Minho (CEICSH 156/2022). Following ethical approval, the main researcher contacted 107 lower and upper public secondary schools in Northern Portugal (e.g. Porto and Braga geographical areas) by email and/or telephone to describe the study and obtain permission to conduct data collection with students. Six primary and two secondary schools agreed to collaborate on this project. Following institutional authorisation, the researcher visited the participating schools and introduced the study to potential participants during scheduled classroom sessions. These presentations were conducted in small classroom groups, typically corresponding to intact classes, and followed a standardised script to ensure consistency across settings. During these sessions, the researcher explained the study objectives, procedures, and ethical safeguards, and allowed time for students to ask questions. To minimise the potential for undue influence on participation, all students were explicitly informed that participation was entirely voluntary and that they could decline or withdraw from the study at any point without negative consequences. At the end of the presentation, the main researcher provided all students with informed consent forms addressed to their parents.

Data collection took place between September 2023 and January 2024 in a classroom previously designated by the school, adolescents interested in participating in the study were required first to present the informed consent form signed by their parent or legal guardian. Subsequently, they read the participant-directed informed assent form and if they agreed to participate voluntarily data collection was conducted through an online questionnaire (using a computer or mobile phone) which participants completed with the researcher present. Questionnaire completion required an average of 40 min.

### 7.2. Data analysis

The CFA and analyses testing invariance across sex were conducted using the software R (v. 4.3.2; R Core Team, 2023). All other data analyses were conducted using IBM SPSS version 27 (IBM Corp, 2020). Descriptive statistics were calculated to characterise the study variables. Psychometric analyses of skewness and kurtosis [*sk* <|3| and *ku* <|10|] were conducted to evaluate the normality of the distribution of responses for SCSQs item (Kline, 2023). Violations of the homogeneity of variance assumption were tested using Levene’s test (Brown & Forsythe, 1974).

After analysing descriptive statistics, we tested four models using CFA, based on previous literature (e.g. Kazlauskas et al., 2020). Model 1 is a one-factor model where the 15 items in the SCSQ loaded onto a DTD latent factor. Model 2 is a correlated three factor model (cluster B, C, and D). Model 3 is a second-order model in which Clusters B, C, and D are modelled as first-order latent factors that load onto a single higher-order DTD factor. Model 4 is a hybrid four factor solution modelled on the DSM-5 PTSD 4 symptom domains (see Ford et al., 2018; Figure 1). These CFA models were specified and estimated using the robust maximum likelihood estimation (MLR; Yuan & Bentler, 2000). The MLR estimator was appropriate given the ordinal nature of the data and in order to adjust for deviations from normality. Model fit was assessed using the chi-square test, the root-mean-square error of approximation (RMSEA; Steiger, 1990), the comparative fit index (CFI; Bentler, 1990), the Tucker Lewis index (TLI; Tucker & Lewis, 1973), the standardised root mean square residual (SRMR; Jöreskog & Sörbom, 1996) indices, the Akaike information criterion (AIC), the Bayesian information criterion (BIC), and the Sample-size adjusted Bayesian information criterion (SABIC). RMSEA and SRMR values of 0.08 and below, CFI and TLI values above 0.90, and the chi square adjustment adequacy test, where the null hypothesis are indicators of good adjustment (Kline, 2023). Lower values of AIC, BIC, and SABIC indicate better relative model fit (Burnham & Anderson, 2004). However, the chi-square test is extremely sensitive to the size of the sample. Specifically, in large samples (i.e. larger than 200), the test result tends to reject the null hypothesis (Hooper et al., 2008). Therefore, the relative chi-square test (i.e. χ^2^/*df*; Wheaton et al., 1977), in which a value lower than two is indicative of good adjustment (Tabachnick & Fidell, 2013), was used in the present study.

The best-fitting model derived from CFA allowed to evaluate the measurement invariance across sex. To verify the evidence of measurement invariance of the SCSQ, the adjustment of the model to the different groups is compared simultaneously, considering different degrees of invariance that include restrictions in increasing complexity: configurational invariance (same factorial structure in all groups, no restrictions); measurement invariance (same statistic of factorial weights); and structural invariance (factor covariances are constrained). When factor weights do not differ significantly between groups, the model is assumed to have weak measure invariance or metric invariance. If the factorial weights and the intercepts do not differ significantly between the groups, the model has strong measure invariance or scalar invariance (Marôco, 2010). To evaluate whether adding equality constraints significantly worsened model fit, we applied the robust Satorra–Bentler scaled chi-square difference test, appropriate for MLR estimation.

The internal consistency was estimated for the three subscales of the SCSQ. Cronbach’s alpha coefficient was used. Good internal consistency is shown when Cronbach’s alpha is higher than 0.70 (Field 2024). In order to verify whether parametric and nonparametric tests of convergent and discriminant validity yielded similar results (Field, 2024), Pearson (parametric) and Spearman (nonparametric) correlation analyses were conducted and found to provide comparable results. Therefore, only results of Pearson correlation analyses are presented.

## 8. Results

### 8.1. Descriptive statistics

Means, standard deviations, range, and intercorrelations of the study variables are presented in Table 1. Participants presented a mean score of 18.63 (*SD* = 10.88; range = 0–45) on the total SCSQ scale, indicating the severity of DTD symptoms. For CPTSD symptom severity, the mean total score on the ITQ-CA scale was 14.57 (*SD* = 11.35; range = 0–47), indicating the severity of CPTSD symptoms. For PTSD symptom severity, the mean total score on the CPSS-5 was 16.83 (*SD* = 17.59; range = 0–77), out of a maximum of 80 points. Emotional dysregulation, as measured by the DERS, showed a mean total score of 2.37 (*SD* = 1.11; range = 1–5). Regarding resilience, participants obtained a mean total score of 106.67 (*SD* = 22.98; range = 28–140).

**Table 1.** Descriptive statistics of the 15 items that make up the SCSQ.

	Total Sample				Males				Females					
Items	Mean	*SD*	Skewness	Kurtosis	Mean	*SD*	Skewness	Kurtosis	Mean	*SD*	Skewness	Kurtosis	Male vs. Female	*p*
B1	1.66	1.04	−.08	−1.21	1.25	1.00	.39	−.87	1.96	.97	−.42	−.99	−7.57	<.001
B2	1.66	1.13	−.20	−1.35	1.24	1.08	.35	−1.16	1.97	1.06	−.65	−.84	−7.19	<.001
B3	1.42	1.11	.11	−1.34	1.12	1.08	.55	−.97	1.65	1.09	−.20	−1.26	−5.12	<.001
B4	1.66	1.11	−.17	−1.33	1.13	1.05	.49	−.97	2.06	.98	−.66	−.72	−9.67	<.001
C1	1.60	1.15	−.10	−1.43	1.09	1.09	.56	−1.01	1.96	1.06	−.60	−.93	−8.48	<.001
C2	.49	.85	1.65	1.67	.58	.91	1.49	1.11	.43	.79	1.78	2.17	1.75	.080
C3	.61	.98	1.40	.61	.52	.93	1.73	1.75	.68	1.01	1.21	.05	−1.74	.083
C4	.74	1.05	1.07	−.36	.44	.89	1.87	2.13	.96	1.12	.67	−1.05	−5.48	<.001
C5	1.20	1.22	.41	−1.43	.94	1.11	.78	−.83	1.40	1.26	.15	−1.63	−4.07	<.001
D1	1.40	1.22	.14	−1.56	.94	1.12	.77	−.88	1.74	1.18	−.30	−1.43	−7.28	<.001
D2	1.83	1.14	−.42	−1.26	1.47	1.15	.06	−1.42	2.10	1.06	−.84	−.61	−5.87	<.001
D3	1.54	1.19	−.05	−1.5	1.04	1.09	.57	−1.06	1.91	1.13	−.53	−1.18	−8.15	<.001
D4	.60	.83	1.18	.40	.55	.79	1.25	.64	.63	.86	1.13	.24	−1.06	.292
D5	1.01	1.13	.64	−1.07	.62	.94	1.36	.71	1.30	1.17	.22	−1.45	−6.77	<.001
D6	1.23	1.14	.33	−1.33	.93	1.04	.74	−.76	1.46	1.16	.05	−1.45	−5.10	<.001
DTD-Total	18.63	10.88	.20	−.99	13.85	9.93	.73	−.42	22.23	10.17	−.09	−.80	−8.68	<.001
DTD-Cluster B	6.40	3.67	−.04	−1.14	4.73	3.39	.51	−.62	7.65	3.36	−.42	−.84	−9.03	<.001
DTD-Cluster C	4.63	3.61	.63	−.42	3.57	3.37	1.09	.44	5.43	3.58	.38	−.53	−5.57	<.001
DTD-Cluster D	7.60	4.65	.15	−.99	5.56	4.19	.62	−.38	9.15	4.38	−.16	−.85	−8.72	<.001

Note: DTD = Developmental trauma disorder; SCSQ = Stress Coping Strategies Questionnaire; *SD* = Standard Deviation.

Regarding exposure to developmental trauma (Table 2), 50.7% of participants (*n* = 225) met both A1 and A2 DTD criteria. An additional 41.9% (*n* = 186) met only A1, while 7.4% (*n* = 33) met only A2. Thus, the majority of the sample (over 50%) fulfilled the full DTD trauma exposure criterion, with most remaining participants endorsing at least partial exposure to developmental trauma. Participants reported a mean of 5.91 (*SD* = 3.56) potential traumatic events, ranging from one to 19 events.

**Table 2.** Descriptive statistics and group comparisons for total Developmental Trauma Disorder (DTD) symptom scores by trauma exposure type.

	*N*	Mean	Standard Deviation	Minimum	Maximum
A1 + A2	225	22.31	10.81	1.00	45.00
A1 only	186	14.94	9.39	0.00	38.00
A2 only	33	14.33	10.87	0.00	34.00

Note: A1 = DTD Criterion A1 (interpersonal victimisation); A2 = DTD Criterion A2 (caregiver-related attachment disruption).

Exposure to one traumatic event was reported by 20 (4.5%) participants, two traumatic experiences were reported by 36 (8.1%) participants, three traumatic experiences were reported by 64 (14.4%) participants, four traumatic experiences were reported by 66 (14.9%) participants, five traumatic experiences were reported by 58 (13.1%) participants, and ≥ 6 experiences were reported by 200 (45.3%) participants.

The most reported traumatic events were: ‘Witnessed a serious accident (not involving self) where someone was seriously injured or killed’ (63.3%), ‘Someone close was seriously injured or ill and almost died or was hospitalized’ (62.6%), and ‘Witnessed non-family members physically fighting or attacking each other’ (61.7%). Frequency of exposure to each traumatic event is presented in Table 3.

**Table 3.** Frequency of exposure to potentially traumatic events.

Trauma event	Total (*N* = 444)	Male (*n* = 191)	Female (*n* = 253)
1.1 Was in a very serious accident where someone could have been (or was actually) seriously injured or killed	135 (30.4%)	65 (34%)	70 (27.7%)
1.2 Witnessed a serious accident (not involving self) where someone was seriously injured or killed	281 (63.3%)	116 (60.7%)	165 (65.2%)
1.3 Experienced a natural disaster where someone was seriously injured or killed or family had to leave home	51 (11.5%)	23 (12%)	28 (11.1%)
1.4 Attacked by a dog or other animal	182 (41%)	82 (42.9%)	100 (39.5%)
1.5a Someone close was seriously injured or ill and almost died or was hospitalised	278 (62.6%)	110 (57.6%)	168 (66.4%)
1.5b Someone close died (not due to old age/natural causes)	151 (34%)	54 (28.3%)	97 (38.3%)
1.6 Was seriously ill and thought might die or required emergency surgery while unconscious	77 (17.3%)	29 (15.2%)	48 (19%)
1.7 Separated from someone loved and depended on for several days (e.g. foster care, detention, war, hospitalization)	99 (22.3%)	35 (18.3%)	64 (25.3%)
1.8 Someone close attempted suicide or serious self-harm	155 (34.9%)	37 (19.4%)	118 (46.6%)
2.1 Physically attacked (e.g. hit, pushed, choked, burned, or attacked with weapon)	99 (22.3%)	44 (23%)	55 (21.7%)
2.2 Threatened with serious harm or death	95 (21.4%)	42 (22%)	53 (20.9%)
2.3a Robbed or approached with intent to rob using threat/intimidation	36 (8.1%)	18 (9.4%)	18 (7.1%)
2.3b Witnessed robbery of a family member or important person	86 (19.4%)	39 (20.4%)	47 (18.6%)
3.1 Witnessed non-family members physically fighting or attacking each other	274 (61.7%)	113 (59.2%)	161 (63.6%)
3.2 Witnessed gunfire or violent attacks at school or in neighbourhood	58 (13.1%)	24 (12.6%)	34 (13.4%)
4.1a Witnessed physical violence between family members	82 (18.5%)	28 (14.7%)	54 (21.3%)
4.1b Witnessed use of weapons between family members	4 (0.9%)	0 (0%)	4 (1.6%)
4.2 Witnessed threats of serious harm or suicide among family members	42 (9.5%)	14 (7.3%)	28 (11.1%)
4.3 Family member arrested or detained by authorities	70 (15.8%)	31 (16.2%)	39 (15.4%)
4.4a Experienced neglect (e.g. lack of food/water, lived on the street, left alone too young)	18 (4.1%)	6 (3.1%)	12 (4.7%)
4.4b Left with someone who used drugs (e.g. heroin, cocaine, hashish)	17 (3.8%)	9 (4.7%)	8 (3.2%)
4.4c Left alone to care for younger siblings for extended periods	45 (10.1%)	19 (9.9%)	26 (10.3%)
5.1 Forced to witness or engage in sexual behaviours (e.g. touching or being touched sexually)	28 (6.3%)	3 (1.6%)	25 (9.9%)
5.2 Witnessed someone being forced to perform sexual acts	22 (5%)	5 (2.6%)	17 (6.7%)
5.3 Forced to watch pornography	18 (4.1%)	6 (3.1%)	12 (4.7%)
6.1 Repeatedly told they were a bad person	165 (37.2%)	60 (31.4%)	105 (41.5%)
6.2 Repeated threats of abandonment due to being a ‘bad’ person	55 (12.4%)	18 (9.4%)	37 (14.6%)

### 8.2. Psychometric analyses of the SCSQ

The distributional characteristics of the 15 items of the SCSQ are presented in Table 3. Item means ranged from 0.49 (C2) to 1.83 (D2), indicating generally low-to-moderate endorsement across the measure. Standard deviations varied between 0.83 and 1.22, suggesting adequate variability among participants.

Skewness values ranged from –0.42 (D2) to 1.65 (C2). Several items displayed positive skewness – particularly items C2 (1.65), C3 (1.40), C4 (1.07), and D4 (1.18) – indicating that higher symptom responses were less frequently endorsed. Kurtosis values ranged from –1.56 (D1) to 1.67 (C2), reflecting both flattened (negative kurtosis) and moderately peaked (positive kurtosis) distributions across items.

At the scale level, the total SCSQ score showed a mean of 18.63 (SD = 10.88), with cluster scores averaging 6.40 (*SD* = 3.67; Cluster B), 4.63 (*SD* = 3.61; Cluster C), and 7.60 (*SD* = 4.65; Cluster D). Skewness and kurtosis values for these composite scores (skewness = –0.04–0.63; kurtosis = –1.14 to –0.42) indicate no substantial departures from normality at the subscale or total-score levels.

Tests of normality indicated a significant deviation from a normal distribution for the total SCSQ score, Kolmogorov–Smirnov *D*(444) = 0.083, *p* < .001, and Shapiro–Wilk *W*(444) = 0.97, *p* < .001. These results suggest caution in the application of parametric statistical procedures without appropriate corrections or robust estimation methods.

Internal consistency of SCSQ scores was examined using Cronbach’s alpha. The SCSQ scale demonstrated excellent internal consistency (α = .91), indicating a high degree of coherence among the 15 items. Reliability coefficients for the DTD clusters scores were acceptable to good: Cluster B (α = .85), Cluster C (α = .71), and Cluster D (α = .79). The SCSD total score also showed strong positive correlations with the three DTD clusters (*r* = .90–.93, all *p* < .001), and the clusters were strongly intercorrelated (*r* = .73–.76, all *p* < .001). These findings support the internal reliability of the SCSQ overall and of the DTD symptom clusters.

### 8.3. Construct validity

The CFA results are presented in Table 4. Model 1, the unidimensional solution, demonstrated an acceptable but comparatively weaker fit to the data (*χ*²/d*f* = 2.56, RMSEA = .063, 90% CI [.053, .073], CFI = .942, TLI = .933, SRMR = .045).

**Table 4.** Model fit of confirmatory factor analysis models.

Model	*χ*²/d*f*	*χ*² (scaled)	d*f*	RMSEA	90% CI	*p* (RMSEA ≤ .050)	*p* (RMSEA ≥ .080)	CFI	TLI	SRMR	AIC	BIC	SABIC
Model 1 (One-factor)	2.56	230.53	90	.063	[.053, .073]	.017	.002	.942	.933	.045	17406.27	17590.59	17447.78
Model 2 (3 correlated factors)	2.43	211.81	87	.060	[.050, .070]	.053	.001	.949	.939	.043	17389.74	17586.34	17434.01
Model 3 (2nd-order)	2.43	211.81	87	.060	[.050, .070]	.053	.001	.949	.939	.043	17389.74	17586.34	17434.01
Model 3 (2nd-order, fixed C variance)	2.40	211.59	88	.060	[.049, .070]	.062	< .001	.949	.940	.043	17387.75	17580.26	17431.10
Model 4 (Hybrid 4-factor)	2.50	209.98	84	.061	[.051, .072]	.036	.001	.949	.936	.042	17393.20	17602.08	17440.23

Note: *χ*² = Chi-square test statistic; d*f* = degrees of freedom; RMSEA = root mean square error of approximation; 90% CI = 90% Confidence Interval for RMSEA; *P*-value H_0_: RMSEA ≤ 0.050 = *p*-value testing the null hypothesis that RMSEA is less than or equal to 0.05; *p*-value H₀: RMSEA ≥ 0.080 = *p*-value testing the null hypothesis that RMSEA is greater than or equal to 0.08; CFI = Comparative Fit Index; TLI = Tucker–Lewis Index; SRMR = standardized root mean square residual; AIC = Akaike information criterion; BIC = Bayesian information criterion; SABIC = Sample-size Adjusted Bayesian Information Criterion. ** p *<* .01; ***p *<* *.001.*

Introducing domain-specific structure improved model performance. Model 2, comprising three correlated first-order factors, showed better fit relative to Model 1, with reductions in *χ*² and improved incremental fit indices (*χ*²/d*f* = 2.43, RMSEA = .060, 90% CI [.050, .070], CFI = .949, TLI = .939, SRMR = .043).

A comparable level of model fit was observed for the second-order model (Model 3), in which the three first-order factors loaded onto a higher-order DTD construct (*χ*²/d*f* = 2.43, RMSEA = .060, 90% CI [.050, .070], CFI = .949, TLI = .939, SRMR = .043). During estimation, the residual variance of factor C approached zero and produced signs of a potential Heywood case, indicating that this factor was almost entirely explained by the higher-order construct. To address this estimation instability and improve parsimony, a modified second-order model was tested in which the residual variance of factor C was fixed to zero (Model 3, fixed C variance; Kolenikov & Bollen, 2012). This constraint did not adversely affect model fit, yielding nearly identical indices (*χ*²/d*f* = 2.40, RMSEA = .060, 90% CI [.049, .070], CFI = .949, TLI = .940, SRMR = .043).

The hybrid four-factor model (Model 4) also demonstrated good fit (*χ*²/d*f* = 2.50, RMSEA = .061, 90% CI [.051, .072], CFI = .949, TLI = .936, SRMR = .042); however, information criteria (AIC, BIC, SABIC) were not superior to those of the three-factor correlated and second-order models.

Taken together, these comparisons indicate that the three-factor correlated model and the second-order model provide the most parsimonious and best-fitting representations of the DTD symptom structure. Given their statistical equivalence and the theoretical coherence of a hierarchical organisation of DTD symptoms, the second-order model with the residual variance of factor C fixed to zero (Model 3_fixed) was retained as the final measurement model.

### 8.4. Measurement invariance across sex

Measurement invariance across sex was assessed using a sequential model comparison procedure involving increasingly constrained nested models: configural, metric, and scalar invariance. Fit indices and chi-square difference tests are presented in Table 5. The analyses were conducted using the best-fitting measurement model, namely the second-order model with the residual variance of factor C fixed to zero (Model 3_fixed).

**Table 5.** Measurement invariance across sex: model fit indexes.

Model	*χ*²	d*f*	CFI	TLI	RMSEA	RMSEA 90% CI	SRMR	AIC	BIC	ΔCFI	ΔRMSEA	ΔSRMR	*p*-value
Configural	303.48	176	.939	.927	.057	[.047, .067]	.049	17228.95	17613.96	–	–	–	–
Metric	334.64	190	.930	.923	.059	[.049, .068]	.068	17233.45	17561.11	.009	.002	.019	.004
Scalar	401.18	201	.904	.899	.067	[.058, .076]	.078	17285.72	17568.33	.026	.008	.010	<.001

Note: d*f* = degrees of freedom; AIC = Akaike Information Criterion; BIC = Bayesian Information Criterion; *χ*² = Chi-square test statistic; Δ*χ*² = chi-square difference between nested models; Δd*f* = difference in degrees of freedom between nested models; *p* = *p*-value associated with the chi-square difference test; CFI = Comparative Fit Index; RMSEA = Root Mean Square Error of Approximation; SRMR = Standardized Root Mean Square Residual; ΔCFI = difference in CFI between models; ΔRMSEA = difference in RMSEA between models; ΔSRMR = difference in SRMR between models.

^a^ Intercepts of items C1 and C2 were freely estimated across sex groups.

Before testing structural invariance, independent-samples *t*-tests were conducted to compare SCSQ symptom levels across sex (Table 1). Overall, females reported significantly higher levels of DTD symptoms than males across most items and composite scores. Specifically, females scored higher on all B and D cluster items, as well as on C1, C4, and C5 (all *p* < .001), with effect sizes ranging from small to large across comparisons.

In contrast, no statistically significant sex differences were found for items C2 and C3 (*p* = .080 and *p* = .083, respectively), and item D4 (*p* = .292), indicating comparable levels between males and females on these specific indicators.

At the aggregate level, females also reported significantly higher total DTD symptom scores compared to males (*p* < .001), as well as higher scores across all three symptom clusters (B, C, and D; all *p* < .001).

The configural model, which imposed no equality constraints across groups, demonstrated acceptable fit, *χ*²(176) = 303.48, CFI = .939, RMSEA = .057 [.047, .067], SRMR = .049. These results indicate that the same latent structure is replicated across male and female adolescents, supporting the assumption that the underlying construct is conceptualised similarly across sex groups.

Constraining factor loadings to equality across groups produced only a modest reduction in model fit. Fit changes relative to the configural model were ΔCFI = .009, ΔRMSEA = .002, and ΔSRMR = .019. The *χ*² difference test was statistically significant, Δ*χ*²(14) = 31.68, *p* = .004; however, consistent with recommendations emphasising approximate fit indices over *χ*² differences in large samples, the magnitude of the changes in fit indices supports the assumption of metric invariance. These findings support the equivalence of factor loadings across sex.

Introducing equality constraints on item intercepts resulted in a more pronounced decline in fit. The scalar model showed *χ*²(201) = 401.18, and comparisons against the metric model yielded ΔCFI = .026, exceeding the ΔCFI ≤ .010 guideline for acceptable invariance, although ΔRMSEA (.008) and ΔSRMR (.010) remained within acceptable ranges. The robust Satorra–Bentler chi-square difference test was statistically significant, Δ*χ*²(11) = 64.98, *p* < .001, indicating that full scalar invariance is not supported. In accordance with best practices for partial invariance, and based on evidence from model modification indices, the intercepts of items C1 and C2 were freely estimated across groups (Schmitt & Kuljanin, 2008). This adjustment improved model performance and yielded a partially invariant scalar model.

Taken together, the results provide evidence for configural and metric invariance of the DTD measurement model across sex, with partial support for scalar invariance after freeing two item intercepts.

### 8.5. Convergent and discriminant validity

Convergent and discriminant validity were examined using Pearson correlations based on the structure supported by the best-fitting CFA model (Model 3, fixed C variance), in which a higher-order DTD factor is represented by three first-order clusters (B, C, D). These results are presented in Table 6.

**Table 6.** Pearson correlations among study variables.

	DTD Total	Cluster B	Cluster C	Cluster D	Trauma exposure	CPTSD	PTSD	DERS	Resilience
DTD Total	–								
Cluster B	.90***	–							
Cluster C	.90***	.73***	–						
Cluster D	.93***	.76***	.75***	–					
Trauma exposure	.49***	.39***	.50***	.50***	–				
CPTSD	.77***	.72***	.68***	.70***	.52***	–			
PTSD	.74***	.69***	.68***	.68***	.53***	.84***	–		
DERS	.65***	.59***	.62***	.59***	.36***	.67***	.66***	–	
Resilience	−.32***	−.32***	−.30***	−.26***	−.24***	−.29***	−.30***	−.14**	–

Note*.* DTD = Developmental trauma disorder; Cluster B = Emotion/somatic; Cluster C = Attentional/behavioural; Cluster D = Interpersonal/self-dysregulation; CPTSD = Complex posttraumatic stress disorder; PTSD = Posttraumatic stress disorder; DERS = Difficulties in emotion regulation.

**p < .01; ***p < .001.

### 8.6. Convergent validity

The SCSQ total score demonstrated moderate-to-strong associations with related trauma-based constructs. Specifically, correlations with CPTSD total symptoms (*r* = .77, *p* < .001) and PTSD symptoms (*r* = .74, *p* < .001) were substantial. DTD total score was also strongly associated with emotion dysregulation (*r* = .65, *p* < .001) and demonstrated a moderate positive correlation with cumulative trauma exposure (TESI total score), *r* = .49, *p* < .001. All DTD cluster scores showed moderate associations with these trauma-based constructs (see Table 6).

As a further test of convergent validity, we examined whether DTD symptoms differed by type of trauma exposure, participants were classified into three groups according to DTD Criterion A: A1 + A2 (interpersonal victimisation plus caregiver-related attachment disruption), A1 only, and A2 only. A Welch one-way ANOVA revealed a statistically significant effect of trauma exposure type on total DTD symptom scores, Welch’s *F*(2, 88.54) = 29.36, *p* < .001, *ω*² = .114, 95% CI [.061, .169]. Post hoc Games–Howell comparisons indicated that adolescents exposed to both A1 and A2 events reported significantly higher DTD symptom levels (*M* = 22.31, *SD* = 10.81) than those exposed to A1 only (*M* = 14.94, *SD* = 9.39; *p* < .001) and A2 only (*M* = 14.33, *SD* = 10.87; *p* < .001). No significant difference was observed between the A1-only and A2-only groups (*p* = .95, see Table 2).

### 8.7. Discriminant validity

Resilience showed a moderate negative association with DTD total symptoms (*r* = −.32, *p* < .001), as well as with DTD cluster B (*r* = −.32, *p* < .001), cluster C (*r* = −.30, *p* < .001), and cluster D (*r* = −.26, *p* < .001).

To formally examine discriminant validity, we compared the magnitude of the correlation between DTD and resilience with those between DTD and trauma-related constructs (CPTSD, PTSD, and emotion dysregulation) using Steiger’s (1980) test for dependent correlations. Results indicated that the association between DTD and resilience was significantly weaker than the associations with CPTSD (*r* = .77), *z* = −17.33, *p* < .001, PTSD (*r* = .74), *z* = −16.65, *p* < .001, and emotion dysregulation (*r* = .65), *z* = −15.35, *p* < .001.

These findings provide strong evidence for discriminant validity, indicating that DTD symptoms are more strongly associated with trauma-related psychopathology and dysregulation than with general adaptive functioning.

## 9. Discussion

To our knowledge, no previous study has validated Developmental Trauma Disorder (DTD) using a self-report measure, and no research has examined the factorial validity of a self-report DTD scale in a community adolescent sample or tested its measurement invariance across sex. Previous empirical work has relied primarily on clinician-administered interviews or clinical samples, limiting the ability to examine the dimensional structure of DTD in non-clinical populations and across cultural contexts (Ford et al., 2018, 2022; Morelli & Villodas, 2022). The present study was designed to address these limitations by evaluating the internal structure, reliability, measurement invariance, and construct validity of a self-report measure of DTD symptoms in a large community sample of Portuguese adolescents. Taken together, the findings provide further support for the validity of DTD as a multidimensional construct, support the use of the SCSQ as a self-report measure of DTD symptoms, and indicate that these results were obtained in a highly trauma-exposed but generally well-functioning community sample.

### 9.1. Latent structure of DTD symptoms

Consistent with the theoretical conceptualisation of DTD as a multidomain construct involving affective/physiological, cognitive/behavioural, and self/relational dysregulation (Ford et al., 2018, 2022; van der Kolk, 2005), multidimensional models provided a better fit to the data than a unidimensional model. Both the correlated three-factor model and the second-order model showed acceptable and comparable fit, and both were superior to the single-factor solution. These findings support the assumption that DTD symptoms are organised into distinct but related domains rather than reflecting a single homogeneous dimension of distress.

The second-order model, in which the three symptom clusters loaded onto a higher-order DTD factor, provided a parsimonious representation of the data and was retained as the final model. During estimation, the residual variance of the cognitive/behavioural dysregulation factor approached zero, suggesting that this domain was largely explained by the higher-order construct. Fixing this residual variance to zero resulted in a more stable and theoretically coherent solution without reducing model fit, indicating that the three domains may be strongly integrated within an overarching DTD dimension in community adolescents.

These results are consistent with previous studies using interview-based assessment, which have supported a multidomain structure of DTD symptoms (Ford et al., 2018, 2022), and extend this evidence by demonstrating that a similar structure can be identified using self-report data in a non-clinical sample. The strong loadings of the first-order factors on the higher-order construct suggest that, although the domains are distinguishable, they may reflect a common underlying dysregulation process associated with exposure to developmental trauma.

### 9.2. Measurement invariance across sex

Measurement invariance across sex was examined to determine whether the structure of DTD symptoms is equivalent for male and female adolescents. The results supported configural and metric invariance, indicating that the same factorial structure and factor loadings were present across sex groups. These findings suggest that the construct of DTD is conceptualised similarly in male and female adolescents and that the items contribute to the latent factors in comparable ways.

Scalar invariance was not fully supported; however, partial scalar invariance was achieved after freeing the intercepts of two items. Partial scalar invariance is generally considered sufficient for meaningful comparisons across groups, particularly in psychological constructs that involve subjective emotional and relational experiences. The lack of full scalar invariance may reflect sex differences in the interpretation or endorsement of specific symptoms rather than differences in the underlying construct.

To our knowledge, this is the first study to examine measurement invariance of DTD symptoms across sex. Establishing invariance is important because it allows potential differences or similarities between males and females to be interpreted as reflecting true variation in symptom levels rather than measurement bias. The literature on trauma-related disorders has reported inconsistent findings regarding sex differences in prevalence and symptom expression. Although some studies have found higher rates of trauma-related symptoms (PTSD or CPTSD) among females (Cloitre et al., 2019; Kimerling et al., 2018), more recent reviews suggest mixed results, with several studies reporting no significant sex differences in CPTSD prevalence (Huynh et al., 2025; Lonnen & Paskell, 2024). Comparisons of SCSQ symptom levels between females and males indicated a largely consistent pattern across items, with females reporting higher scores on most indicators. However, this pattern was not uniform, as no significant differences were observed for items C2, C3, and D4, suggesting that some aspects of the construct may be experienced or reported similarly across sex. Taken together, these findings indicate that, although females tend to report higher levels of DTD symptomatology, the overall pattern of symptom endorsement is broadly comparable across groups. This is consistent with the partial scalar invariance observed, suggesting that most items function similarly across males and females, with only limited item-level differences in intercepts. The present findings therefore provide further support for the validity of DTD as a construct applicable to both male and female adolescents, despite evidence of higher overall levels of symptom endorsement among females.

### 9.3. Internal consistency

The self-report DTD scale demonstrated excellent internal consistency for the total score and good internal consistency for the three symptom clusters. These values are comparable to those reported in previous studies using interview-based DTD measures (Ford et al., 2018) and are consistent with reliability estimates commonly observed in trauma-related symptom scales. The results indicate that the items within each domain show adequate coherence while still capturing distinct aspects of dysregulation, supporting the reliability of the scale for use in community adolescent samples and providing further evidence for the validity of the SCSQ as a self-report measure of DTD symptoms.

### 9.4. Convergent validity

Convergent validity was supported by moderate to strong associations between DTD symptoms and related constructs. As expected, DTD scores were strongly correlated with CPTSD and PTSD symptoms, as well as with emotion dysregulation. These findings are consistent with theoretical models proposing that DTD reflects pervasive difficulties in regulation, self-organisation, and relational functioning following chronic interpersonal trauma (D’Andrea et al., 2012; Ford et al., 2018).

DTD symptoms were also moderately associated with cumulative trauma exposure, indicating that greater exposure to potentially traumatic events is related to higher levels of dysregulation. Importantly, adolescents who met both interpersonal victimisation (A1) and caregiver-related attachment disruption (A2) criteria reported significantly higher DTD symptom levels than those exposed to only one type of trauma. This pattern is consistent with the DTD formulation, which emphasises the combined impact of interpersonal trauma and attachment disruption in the development of complex dysregulation.

### 9.5. Discriminant validity

Discriminant validity was supported by the pattern of associations with resilience. DTD symptoms showed moderate negative correlations with resilience, and these associations were lower in magnitude than those observed with trauma-related variables. Although no previous study has examined the relationship between DTD symptoms and resilience, the present findings are consistent with broader trauma literature showing that higher levels of PTSD/CPTSD are associated with lower resilience (Duan et al., 2015; Fernández-Fillol et al., 2024). From a theoretical perspective, resilience reflects adaptive functioning and the ability to regulate emotions and relationships under stress, whereas DTD is characterised by persistent dysregulation across affective, behavioural, and relational domains (Ford et al., 2018; Horn & Feder, 2018). The moderate magnitude of the associations therefore supports the discriminant validity of the construct, suggesting that DTD symptoms are related to, but distinct from, resilience. In future research on discriminant validity, it will be important to determine if DTD as assessed with the SCSQ is associated with measures of dysregulation and with functional impairment (as was found with the DTD interview; Ford et al., 2018, 2022) independently of (i.e. after controlling for the effects of) PTSD, CPTSD, and other forms of psychopathology.

### 9.6. Interpretation of trauma exposure levels

One notable finding was the high level of trauma exposure reported in the present community sample, with a large proportion of participants meeting the DTD exposure criterion – although this is consistent with *findings from other studies* (e.g. McLaughlin et al., 2013) reporting prevalence of trauma exposure in adolescent community samples of >60%. A first possible explanation concerns the characteristics of the trauma history measure used in this study. The TESI includes a relatively broad range of potentially traumatic events and differentiates between direct and indirect exposure, resulting in a larger number of reportable experiences compared to shorter instruments such as the Life Events Checklist, in which several types of exposure may be grouped into a single category. The use of detailed self-report checklists that assess multiple forms of interpersonal and non-interpersonal adversity may therefore lead to higher prevalence estimates, particularly in community samples.

A second explanation relates to differences between assessment methods. Prevalence rates of childhood adversity are known to vary widely depending on the source of information, with official records or informant-based reports typically yielding much lower estimates than retrospective or self-report surveys (Mathews et al., 2020; Mehta et al., 2023). This discrepancy became particularly evident during the COVID-19 pandemic, when studies based on administrative or child protection data often suggested stable or decreasing rates of maltreatment, whereas self-report studies frequently documented increases in exposure to violence, family conflict, and neglect (Klika et al., 2025). Self-report methods are more likely to capture experiences that do not come to the attention of authorities, including changes in parenting practices and family functioning. Several studies conducted during the pandemic reported higher levels of parental stress, increased use of harsh discipline, greater conflict between parents and children, and elevated risk factors for psychological aggression and neglect (e.g. Blumenthal, 2023; Rodriguez et al., 2021; Wang et al., 2024).

At the same time, mean levels of PTSD, CPTSD, and DTD symptoms in the present sample were generally moderate, and resilience scores were relatively elevated, suggesting that the sample represents a highly exposed but overall well-functioning group of adolescents. This pattern may reflect the presence of protective factors that buffer the psychological impact of adversity, as well as the inclusion of events that, although potentially stressful, do not necessarily lead to persistent dysregulation in all individuals. Nevertheless, the high prevalence of trauma exposure should be interpreted cautiously, as it may reflect methodological, contextual, or cultural factors specific to the present study. Future research is needed to determine whether similar rates are observed in other community adolescent samples using comparable assessment procedures.

### 9.7. Limitations and future research

Several limitations should be considered. First, the cross-sectional design prevents conclusions about the developmental course of DTD symptoms or causal relationships between trauma exposure and dysregulation. Second, all measures were based on self-report, which may be influenced by recall bias or response style, and no clinician-administered interview was used to confirm DTD, PTSD, or CPTSD diagnoses. Third, although the use of a large community sample is a strength of the study, the high level of trauma exposure may limit generalizability to other adolescent populations. Fourth, the study was conducted in a Portuguese sample, and further cross-cultural validation is needed to determine whether the same structure is observed in other cultural contexts. Future research should also examine test–retest reliability, longitudinal stability, and clinical validity in treatment-seeking samples.

## 10. Conclusion

The present findings provide support for the validity of DTD as a multidimensional construct that can be assessed using self-report by adolescents. The results indicate that the SCSQ demonstrates a coherent factorial structure, good reliability, acceptable measurement invariance across sex, and expected patterns of convergent and discriminant validity. These findings support the use of the SCSQ as a self-report measure of DTD symptoms and extend previous interview-based research by showing that DTD can be reliably assessed in community samples and across sexes. At the same time, the results should be interpreted in light of the high trauma exposure observed in the sample and the exclusive use of self-report data. Therefore, the scale should be considered appropriate for research and screening purposes rather than as a standalone clinical tool, and further studies in clinical and longitudinal samples are needed to clarify the clinical utility of the SCSQ and the DTD construct.

## Data Availability

The datasets generated and/or analysed during the current study are not publicly available due to ethical and privacy restrictions related to sensitive participant data but are available from the corresponding author on reasonable request.

## Use of generative AI

Generative artificial intelligence (AI), specifically ChatGPT (OpenAI), was used to assist with (a) language editing to improve the clarity and quality of the English writing, and (b) generating initial R code for statistical analyses. The generated code supported the implementation of confirmatory factor analyses and measurement invariance testing. All outputs were carefully reviewed, validated, and adapted by the authors. The authors take full responsibility for the accuracy, integrity, and interpretation of the analyses and results.
